# Shifts in species richness, herbivore specialization, and plant resistance along elevation gradients

**DOI:** 10.1002/ece3.296

**Published:** 2012-07-01

**Authors:** Loïc Pellissier, Konrad Fiedler, Charlotte Ndribe, Anne Dubuis, Jean-Nicolas Pradervand, Antoine Guisan, Sergio Rasmann

**Affiliations:** 1 Department of Ecology and Evolution, University of Lausanne Bâtiment BiophoreCH-1015, Lausanne, Switzerland; 2 Department of Tropical Ecology and Animal Biodiversity, University of ViennaRennweg 14, A-1030, Vienna, Austria

**Keywords:** Diet breadth, generalist herbivores, host plant, phylogenetic ecology, plant resistance, plant–herbivore interaction, polyphagy, specialist herbivores

## Abstract

Environmental gradients have been postulated to generate patterns of diversity and diet specialization, in which more stable environments, such as tropical regions, should promote higher diversity and specialization. Using field sampling and phylogenetic analyses of butterfly fauna over an entire alpine region, we show that butterfly specialization (measured as the mean phylogenetic distance between utilized host plants) decreases at higher elevations, alongside a decreasing gradient of plant diversity. Consistent with current hypotheses on the relationship between biodiversity and the strength of species interactions, we experimentally show that a higher level of generalization at high elevations is associated with lower levels of plant resistance: across 16 pairs of plant species, low-elevation plants were more resistant vis-à-vis their congeneric alpine relatives. Thus, the links between diversity, herbivore diet specialization, and plant resistance along an elevation gradient suggest a causal relationship analogous to that hypothesized along latitudinal gradients.

## Introduction

Darwin (1859) and Wallace (1878) were among the first to document more diverse and specialized biotic interactions, mediated by higher numbers of species, in the tropics compared with higher latitudes. Since then, multiple hypotheses have been developed to explain gradients in the strength of biotic interactions, and how these relate to gradients in species diversity (e.g., Fischer 1960; MacArthur 1972; Pennings et al. 2009; Schemske et al. 2009). Speciation and diversification have been postulated to be catalyzed by stronger and more specialized biotic interaction, through the species-driven expansion of available resources and niche space (Schemske et al. 2009). In particular, high herbivorous insect richness in the tropics has been suggested to shape plant diversification through the evolution of defense specialization (Ehrlich and Raven 1964; Coley and Aide 1991).

Higher herbivore richness and abundance in tropical regions compared with temperate climates may have promoted more efficient plant defenses and the need for insects to specialize to overcome them (Ehrlich and Raven 1964; Levin and York 1978; Coley and Aide 1991). While there is evidence of more intense insect herbivory on plants in the tropics (Pennings et al. 2009; Schemske et al. 2009), there is still conflicting evidence with regard to possible differences of diet breadth (Fiedler 1998; Dyer et al. 2007; Novotny et al. 2007; Slove and Janz 2010) and plant defense (Moles et al. 2011) along latitude, and these dimensions have never been simultaneously assessed.

Other environmental gradients, such as elevation, offer biogeographically controlled means to address the relationships between diversity, herbivore diet specialization, and plant defense. Elevational studies, which encompass steep clines over relatively small geographical ranges, are more resistant to problems of dispersal and historical contingency than latitudinal studies. When ascending rapidly from lowland to alpine environments, species experience strong abiotic variation over extremely short distances (Körner 2003). With increasing elevation, changes in partial pressure, temperature, wind speed, UV exposure, and soil have been shown to affect the phenology, morphology, physiology, and chemistry of host plants (Hodkinson 2005) that in turn may affect defensive capacities. Recent evidence indicates that herbivore diversity (Beck et al. 2011), herbivore attack (Scheidel and Bruelheide 2001), and herbivore abundance and specialization (Rodríguez-Castañeda et al. 2010) also vary along altitudinal gradients, paralleling the findings for latitude.

In this study, we employed hypotheses for latitudinal gradients with elevational gradients by simultaneously investigating variation in diversity and diet breadth in the superfamily Papilionoidea, along with resistance of potential host plants. We postulated that, while coevolution of plant defense and insect resistance may have driven plant and insect diversification in warmer and more stable conditions (i.e., lowlands), the decrease of herbivore abundance with elevation should relax plant resistance and promote insect generalization (Coley and Aide 1991). Specifically, we tested the following hypotheses: (1) due to reduced herbivore pressure and/or more severe environmental conditions, plant resistance against herbivores should be relaxed at high altitudes and (2) lower plant resistance at high elevations should promote greater diet breadth in butterfly species that have colonized colder environments.

## Materials and Methods

### Field sampling of butterflies and plants

**Figure 1:**
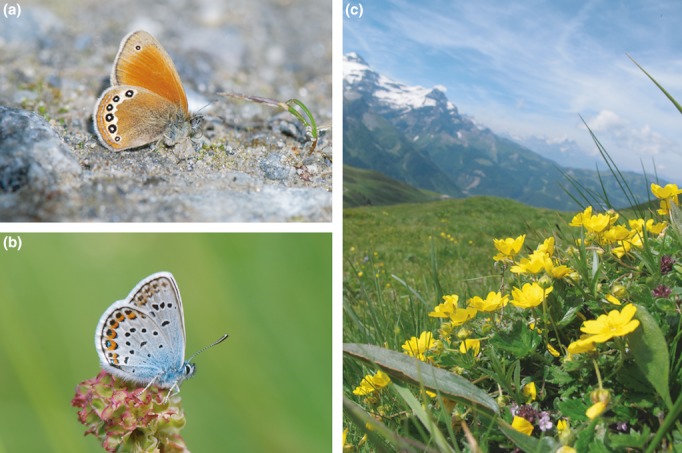
Examples of Alpine biodiversity included in the present study. Shown is (A) the nymphalid butterfly *Coenonympha gardetta*, (B) the lycaenid butterfly *Plebejus argus*, and (C) a sample of the Swiss Alpine flora. In the foreground two species included in the multi-species resistance experiment are visible: *Thymus alpestris* (now *T. pulegioides* subsp. *pulegioides*) with pink flowers, and *Potentilla aurea* with yellow flowers.

Over the course of two consecutive summers, we investigated species composition of butterfly superfamily Papilionoidea (sensu Heikkilä et al. 2012) and angiosperm communities over a >700 km^2^ area of the Western Swiss Alps (Fig. 1). The study area ranges in elevation from 800 m to 3210 m a.s.l. (Fig. S1). We selected sites outside forested areas, following a balanced random-stratified sampling design based on elevation, slope, and aspect (Hirzel and Guisan 2002). To assess butterfly richness and species composition along elevational gradient, we sampled 192 sites between May 15 and September 15, at hours when butterflies were most active (i.e., 10:00–17:00 h) and only in good weather conditions. Each 50 × 50 m site was visited every 3 weeks, during which we conducted 45 min of observation. Butterflies were collected with a net and identified and inventoried by species. We collected adult specimens rather than caterpillars because they were more conspicuous, easier to identify, and more reliable to survey. By excluding migratory species (see below) we assume that the correlation between adult observation and sites reflects one of the larvae. Additionally, within a 4 m^2^ area at the center of each of the 192 sites, we exhaustively inventoried the vegetation to characterize the plant community of the site (for details refer to Dubuis et al. 2011). The relationships between plant richness and butterfly richness and abundance as a function of altitude were analyzed using a General linear model (GLM) with a quasi-Poisson distribution and included both linear and quadratic terms given the nonlinear nature of the relationships.

### Analysis of diet breadth along elevation gradients

We reconstructed the phylogeny of all angiosperm genera and butterfly species within the study area (see Figs. S2, S3, and methodological details of phylogenetic reconstruction therein). The larval host plant affiliations (i.e., plant genera) of each butterfly species were extracted from a comprehensive literature survey (Ebert and Rennwald 1993; Lepidopterologen-Arbeitsgruppe 1994; Huemer 2004). We measured the larval diet breadth of each butterfly species as the mean phylogenetic distance (MPD) between its host plant genera. Although across closely related, coexisting species we might observe overdispersion of secondary metabolite diversity due to herbivore-mediated character displacement (Kursar et al. 2009), phylogenetic distance can be observed as an integration of plant traits, such as chemical defenses, and has been argued as an optimal predictor of insect specialization (Rasmann and Agrawal 2011). The use of phylogenetic distance rather than an arbitrary taxonomic unit (such as number of genera) can also overcome the subjectivity of taxonomic scale choice (Symons and Beccaloni 1999). Moreover, plant species and genus diversity decrease with rising elevation, which could lead to spurious observation of higher degrees of polyphagy at low elevations (Fig. S4). We used the “MPD” function in the Picante R package implementing tools for extracting MPD between host plants (Kembel et al. 2010). We related the species' MPD to mean elevation of occurrence for each species using a linear model (LM), accounting for phylogenetic distance among butterfly species as implemented in the phylogenetic generalized least square (PGLS) in the caper R package (Orme 2011). The PGLS function addresses phylogenetic nonindependence between species by incorporating covariance between taxa into the calculation of estimated coefficients. For this analysis, we considered only nonmigratory butterfly species that occurred in at least 10% of the 192 sampled communities, to avoid less accurate estimates of elevation optima for rare species due to low sample size. Additionally, considering all nonmigratory species, while including all species, we related the elevation of each locality to the corresponding butterfly community mean diet breadth, which was measured as the average value of diet breadth (i.e., MPD) for all butterfly species observed in each community. To include nonlinear relationships, we related average MPD to elevation using a Gaussian GLM with both linear and quadratic terms.

### Plant resistance along elevation gradients

To assess the degree of plant resistance at different elevations, we performed a cafeteria test using *Spodoptera littoralis* caterpillars (obtained from Syngenta, Switzerland) in a phylogenetically controlled paired design. *S. littoralis* is a generalist known to consume plants of at least 40 different families (Brown and Dewhurst 1975) and is widely used for performing plant resistance bioassays. Analysis of congeneric plant species that span large segments of the phylogeny can be understood as a phylogenetically correct independent contrast, as long as the compared branches do not intersect (Felsenstein 1985). We used *S. littoralis* caterpillars, not yet present in Switzerland, as a nonadapted caterpillar to remove the confounding effect of possible local adaptation to plants.

Fresh leaves of 16 pairs of congeneric plant species were sampled at either high (about 2000 m a.s.l) or low (about 800 m a.s.l.) elevation (Table S1). We chose congeneric plant pairs that never occupy the contrasted elevations simultaneously. The leaves were preserved at 5°C before being offered to neonate caterpillars under controlled temperature (24°C day) and light (14/10 h) conditions (*n* = 10 replicates per species) and were changed twice over the course of the 1-week trials. After 7 days, the caterpillar weights were measured.

To remove the confounding effect of leaf toughness, a potential antiherbivore trait (Schoonhoven et al. 2005) that may vary with elevation (Körner 2003), we also determined mean specific leaf area (SLA) for each plant species in the experiment. Between 4 and 20 individuals per plant species were measured, from locations of contrasting exposure, slope, and elevation to cover each plant species' total ecological range and capture its entire regional intraspecific trait variation. For each individual plant, a fully developed leaf was sampled, immediately weighed, and scanned using ImageJ software (http://rsbweb.nih.gov/ij/) to quantify its area. The leaves were then dried at 40°C for one night to obtain dry mass, and SLA was then calculated as the ratio of leaf surface to dry mass, expressed in mm² mg^−1^.

We related the weight of each caterpillar to the elevation and SLA, accounting for the genus of the host plant. We tested the significance using a permutation-based analysis of variance, as implemented in the lmPerm package in R (Wheeler 2010), to overcome nonnormally distributed data, and we used a rank-transformation to overcome heterogeneity of variances. We related the survival (0 or 1) to elevation, accounting for genus using a GLM with binomial distribution.

## Results

### Shifts in diet breadth along elevation gradients

**Figure 2:**
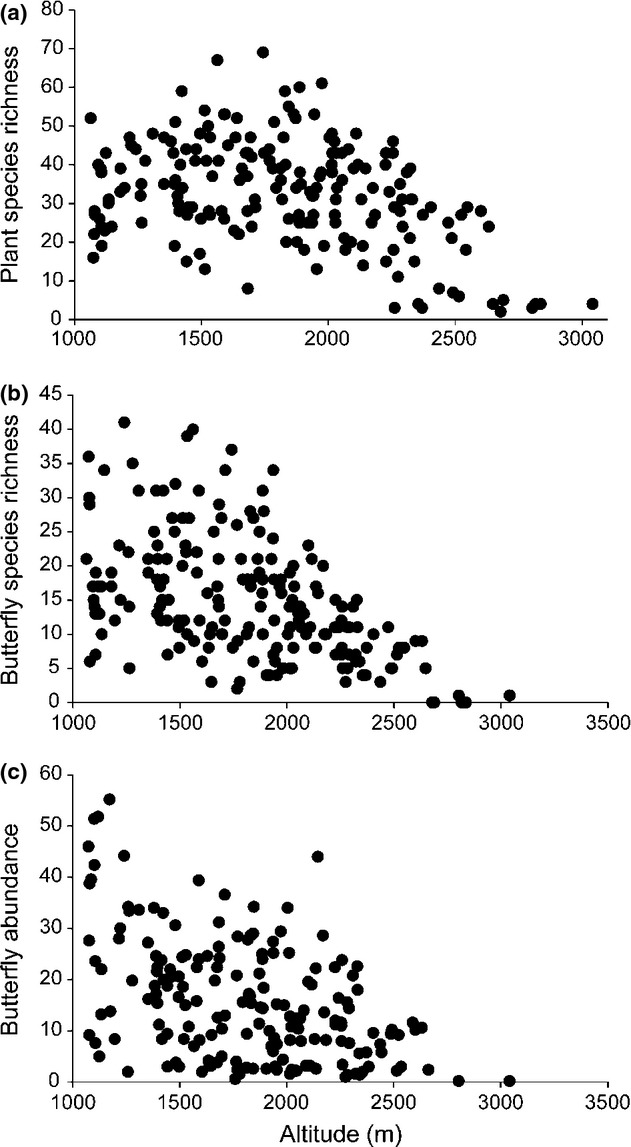
Species richness and abundance along elevation gradients. Shown are the relationships between elevation and (a) plant species richness, (b) butterfly species richness, and (c) butterfly abundance, sampled in 192 plots along elevation gradients of the Swiss Alps.

**Figure 3:**
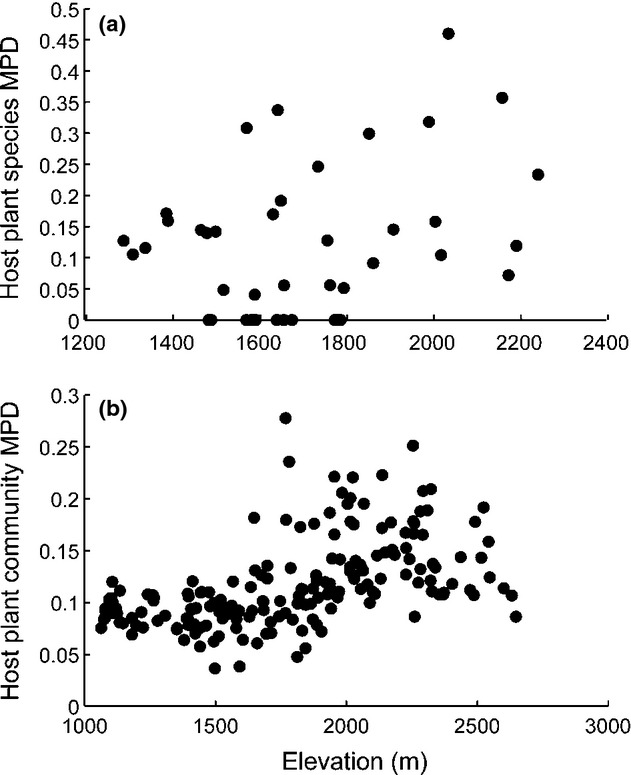
Host specialization along elevation gradients. (a) Relationship between the average elevation of occurrences of the butterfly species and their larval diet breadth, and (b) relationship between the elevation of the sampled butterfly communities and the community mean of the butterfly larval diet breadth.

**Figure 4:**
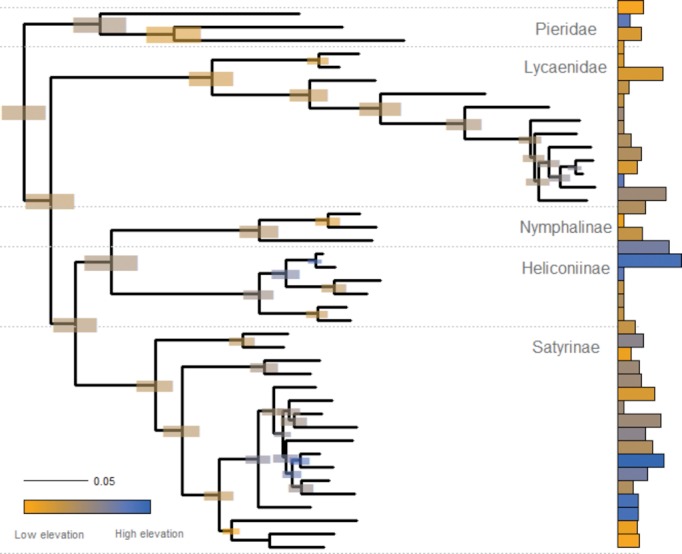
Butterfly phylogeny and their diet breadth in relation to altitude. Shown is the pruned tree of butterfly species that are both nonmigratory and found in at least 10% of the field plots from a comprehensive phylogeny of all butterflies sampled (see Fig. S2). Bars on the right show butterfly diet breadth measured as the mean phylogenetic distance between the host plant species. Each bar is colored according to the optimum habitat for each butterfly species, where colder (blue) colors represent high elevations, and warmer (orange) colors represent low elevations. Ancestral state of elevation optimum was reconstructed with maximum likelihood using the ace function in ape R package and shows that colonization of high elevation is a derived state.

Overall, we found that plant species richness (Fig. 2a, linear: *t* = −7.1, *P* < 0.0001, quadratic: *r* = 0.55, *t* = −7.2, *P* < 0.0001), butterfly species richness (Fig. 2b, linear: *t* = −5.64, *P* < 0.0001, quadratic: *t* = −3.04, *P* < 0.0001), and butterfly abundance (Fig. 2c, linear: *t* = −6.9, *P* < 0.0001, quadratic: *t* = 1.9, *P* = 0.053) all decreased with increasing elevation. We also observed a peak of richness at mid-elevation for plants, which would point to a mid-domain effect as suggested by Grytnes (2003), but this was not the case for butterflies. On the other hand, larval diet breadth increased with increasing elevation (i.e., insects became more generalized) when accounting for phylogenetic nonindependence between butterfly species using a phylogenetic GLS model (Fig. 3a, df = 47, *t* = 2.27, *P* = 0.02). As corollary to this second result, butterfly communities at high elevations were found to be composed of species displaying greater average larval diet breadth compared with low-elevation communities (Fig. 3b, linear: *t* = 6.12, *P* < 0.0001, quadratic: *t* = −3.25, *P* = 0.001). Overall, we found that several clades in the Papilionoidea family have independently adapted to tolerate the severe environmental conditions found at high elevations, and to increase their diet breadth (Fig. 4, blue tones on the phylogeny, along with longer bars).

### Plant resistance along elevation gradients

**Figure 5:**
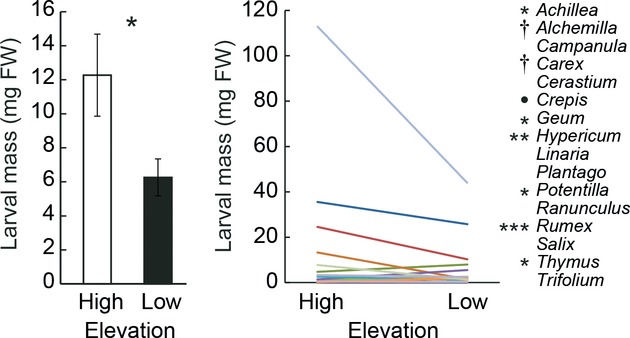
Lower plant resistance for high-elevation plant species. Shown is the mean (± 1SE) *Spodoptera littoralis* larval mass after 7 days of feeding on 16 high elevation (open bars) plant species, and their congeneric low elevation (black bars) across all genera (left), and reaction norms for each individual plant genus (right) (^•^*P* < 0.1; *P < 0.05; **P < 0.01; ***P < 0.001, †, no survival on both species, using permutation analyses on SLA corrected data). All significant effects indicate lower growth rate on low-elevation plants. Plant species were sampled along the phylogeny to include the most commonly found families (Table S1).

Across 16 pairs of congeneric plant species, we found that caterpillars grew 50% less rapidly on low-elevation plants compared with high elevation plants (Fig. 5, permutation analysis; elevation effect, R Mean Sq = 1.53, *P* = 0.009). The analysis took into account potential variation among genera (genus effect, R Mean Sq = 6.4, *P* < 0.0001) and variation in SLA (SLA effect, R Mean Sq = 0.42, *P* = 0.21, and elevation*SLA interaction, R Mean Sq = 0.79, *P* = 0.075) as a measure of leaf thickness. Particularly, high elevation plants had 20% denser leaves, but this result was found to be genus specific. Survival of neonate caterpillars, a critical step in the lepidopteran life-cycle (Schoonhoven et al. 2005), did not differ between the high and low elevations (Fig. S5, *t* = −0.276, *P* = 0.78).

## Discussion

This study follows hypotheses for latitudinal gradients (Beaver 1979; Coley and Aide 1991; Dyer et al. 2007) and found that, along with overall reduced species richness, butterflies occurring at high elevations displayed greater diet breadth, while host plants were less resistant to generalist herbivores. The results from field sampling and empirical bioassays of regional butterfly fauna suggest that lower plant defense at high elevations promotes trophic generalization in those butterfly species that have colonized colder conditions.

Because of the correlative nature of this study, it remains to be tested whether relaxation of plant resistance traits at high elevations is a direct result of lower herbivore pressure, as suggested by the lower abundance of butterflies found in our study, or if plant resistance traits are also, or even primarily, influenced by environmental factors, such as temperature or resource availability. Indeed, a number of plant traits, including foliar nitrogen (Erelli et al. 1998; Hengxiao et al. 1999; Richardson 2004), defensive chemistry (alkaloids, coumarins, phenolics, and terpenes [Erelli et al. 1998; Hengxiao et al. 1999; Salmore and Hunter 2001; Zehnder et al. 2009]), and structural compounds, such as lignin and cellulose (Richardson 2004), vary with elevation, which may result from a combination of herbivory pressure and abiotic conditions. Leaves with higher concentrations of cellulose and lignin are stronger, more resistant to damage, and potentially longer lived than leaves with lower fiber concentrations (Abrahamson et al. 2003; Richardson 2004). Similarly, phenolic compounds can protect leaves from photodamage by acting as antioxidants and reducing herbivore fitness (Close and McArthur 2002).

An analysis of soil nutrient elements (C/N, P) in the same study area showed no relationship with elevation (Fig. S6). On the other hand, steep altitudinal gradients are regularly associated with a reduction in temperature (Fig. S7). Lower temperatures are generally associated with slow-growing conditions (Körner 2003), and in accordance with a classic hypothesis, slow-growing plants should invest more in defenses (Coley et al. 1985; Fine et al. 2004). However, we argue that this should be true only in the case of identical abiotic conditions and probability of herbivore attack. We propose that, along elevation gradients, even if plants have slower growth rates (Körner 2003), lower herbivore abundance leads to relaxation of plant defenses, which in turn favors increased herbivore diet breadth. Lower temperatures may also reduce photosynthetic capacity and therefore the resources that can be invested in defense. Nonetheless, more empirical data on leaf damage, herbivore pressure, and insect communities that have colonized colder environments are needed to test whether herbivores and/or abiotic conditions are the main drivers of plant defense evolution.

Other processes may have also shaped the observed pattern of less herbivore specialization at high altitudes. Climatic oscillations that spanned the Quaternary may have reduced the number of species-specific plant-insect interactions in alpine habitats by limiting the timescale over which long-term coevolutionary processes could occur, due to recurrent local extinctions and continually shifting available host plant ranges (Schönswetter et al. 2005). Herbivorous insects, unable to follow these shifting ranges, would have become locally extinct if no other available feeding plants were present, thus favoring the emergence of generalist habits. However, this would only be true for herbivores with very low dispersal capacity. Alternatively, at high elevations, short-lived exothermic insects need to distribute their eggs during the short periods of good flight weather. The less selective they are, the easier they can achieve high fecundity. At high elevation, we observed a few rare species (observed at <10% of the sites) that display a restricted trophic niche (e.g., *Plebejus glandon*, *Erebia pluto*, *E.mnestra*, *Pontia callidice* feeding on closely related *Androsace* spp., Poaceae and Brassicaceae, respectively). This suggests that although relaxed plant defenses at high elevation may facilitate the acquisition of broader trophic niche breadth, remaining specialized is still an option. However, the low ecological success indicated by the rarity may be explained by their specialization in the unstable environment at high elevation. Finally, other biotic factors, such as top-down effects from predators, have been shown to contribute to insect feeding habits (Singer et al. 2012) and in turn to plant-defense expression. These factors should thus be included in future experiments on specialization along elevation gradients (Preszler and Boecklen 1996).

Can our results be generalized to other ecosystems? Novotny et al. (2005) found no difference in moth diet breadth between sites from two elevations at 200 and 1800 m a.s.l. in tropical New Guinea, while Rodríguez-Castañeda et al. (2010) showed a decrease in moth specialization with elevation in Costa Rica and Ecuador, suggesting site-specific patterns. Interestingly, Papilionoidea, which generally show high levels of diet specialization compared with other Lepidopteran clades, do not seem to be the only insect group that displays more specialized species in the lowlands of the Alps. A survey of recent literature shows that bees (Apiformes) and wood-boring beetles (Buprestidae) also display this pattern of greater diet breadth with increasing altitude (Rasmann et al. In press), indicating that generalization at high elevation might be taxonomically widespread.

In conclusion, our study provides evidence that lower plant defense at high elevations, caused by relaxed biotic pressure, promoted increased butterfly diet breadth. Reciprocal selective responses between plants and insects are one major driver of current terrestrial diversity, and the evolution of plant defensive traits has been suggested to sculpt such patterns (Ehrlich and Raven 1964). Capturing mechanisms of plant–insect interactions along elevation gradients, as performed in our study, allows for better predictions of ecosystem modification during climate change, including how herbivores that are migrating to high elevations ahead of the vegetation might behave when they encounter novel and less resistant potential host plants.

## Supporting Information

**Table S1.** A description of the 32 plant species used in the bioassay.

**Figure S1.** Study area in the Western Swiss Alps.

**Figure S2.** Angiosperm phylogeny of all plants genera sampled along the elevation gradient.

**Figure S3.** Butterfly phylogeny of all species sampled along the elevation gradient.

**Figure S4.** Diet breath along elevation gradients using plant genera.

**Figure S5.** Insect survival on high- and low-elevation plant species.

**Figure S6.** Soil nutrient composition along elevation gradients.

**Figure S7.** Degree-days along elevation gradients.
