# Supplementary material for: Shifts in species richness, herbivore specialization, and plant resistance along elevation gradients
Source: Ecol Evol. 2012 Jul 1;2(8):1818–25. doi: 10.1002/ece3.296 (PMC3433986; doi:10.1002/ece3.296)
Supplement: Supplementary file 1 [file ece30002-1818-SD1.doc]

Supplementary Information for:

Ecological gradients: species richness, insect specialization and plant resistance

Loïc Pellissier, Konrad Fiedler, Charlotte Ndribe, Anne Dubuis, Jean-Nicolas Pradervand, Antoine Guisan, and Sergio Rasmann

**SUPPORTING INFORMATION**

The following Supporting Information is available for this article:

**Table S1.** A description of the 32 plant species used in the bioassay.

**Figure S1.** Study area in the Western Swiss Alps.

**Figure S2.** Angiosperm phylogeny of all plants genera sampled along the elevation gradient.

**Figure S3.** Butterfly phylogeny of all species sampled along the elevation gradient.

**Figure S4.** Diet breath along elevation gradients using plant genera.

**Figure S5.** Insect survival on high and low elevation plant species.

**Figure S6**. Soil nutrient composition along elevation gradients.

**Figure S7**. Degree-days along elevation gradients.

**Table S1.** Resistance of high and low elevation plant species. Shown is the mean specific leaf area (SLA), the larval weight and survival on 16 high elevation species and their 16 congeneric low elevation species. Plant species were chosen to cover an important proportion of the angiosperm phylogeny found throughout the Swiss Alps.

| Family | Genus | Species | Habitat | SLA (mm² mg-1) | Larval weight (mg) | % survival |
| --- | --- | --- | --- | --- | --- | --- |
| Rosaceae | *Achillea* | *atrata* | high | 21.25 | 35.5608 | 0.9 |
|  |  | *millefolium* | low | 28 | 25.7595 | 0.8 |
| Rosaceae | *Alchemilla* | *conjuncta* | high | 15.56 | 0 | 0 |
|  |  | *vulgaris* | low | 23.45 | 0 | 0 |
| Campanulaceae | *Campanula* | *scheuchzeri* | high | 21.94 | 4.7507 | 0.6 |
|  |  | *patula* | low | 34.89 | 7.9632 | 0.3 |
| Cyperaceae | *Carex* | *sempervivens* | high | 9.7 | 0 | 0 |
|  |  | *sylvatica* | low | 30.09 | 0 | 0 |
| Caryophyllaceae | *Cerastium* | *latifolium* | high | 25.65 | 0.214 | 0.1 |
|  |  | *fontanum* | low | 28.85 | 1.7605 | 0.4 |
| Asteraceae | *Crepis* | *aurea* | high | 30.84 | 13.2896 | 0.4 |
|  |  | *foetida* | low | 26.86 | 1.4664 | 0.4 |
| Rosaceae | *Geum* | *montanum* | high | 15.5 | 0.4003 | 0.4 |
|  |  | *urbanum* | low | 38.46 | 0 | 0 |
| Hypericaceae | *Hypericum* | *maculatum* | high | 24.88 | 24.5439 | 1 |
|  |  | *perforatum* | low | 26.06 | 10.2522 | 0.9 |
| Scrophulariaceae | *Linaria* | *alpina* | high | 20.47 | 1.0511 | 0.3 |
|  |  | *vulgaris* | low | 19.98 | 2.6182 | 0.3 |
| Plantaginaceae | *Plantago* | *alpina* | high | 16.17 | 1.4289 | 0.2 |
|  |  | *lanceolata* | low | 25.65 | 5.5203 | 0.8 |
| Rosaceae | *Potentilla* | *aurea* | high | 22.98 | 2.586 | 0.8 |
|  |  | *repens* | low | 25.1 | 1.119 | 0.7 |
| Ranunculaceae | *Ranunculus* | *alpestris* | high | 20.54 | 0.206 | 0.3 |
|  |  | *acris* | low | 26.42 | 2.0108 | 0.5 |
| Polygonaceae | *Rumex* | *alpinus* | high | 32.25 | 112.9689 | 1 |
|  |  | *obtusifolius* | low | 31.04 | 43.9022 | 1 |
| Salicaceae | *Salix* | *repens* | high | 15.09 | 0.4888 | 0.1 |
|  |  | *triandra* | low | 11.61 | 0.3165 | 0.1 |
| Lamiaceae | *Thymus* | *alpestris* | high | 16.19 | 7.7454 | 0.7 |
|  |  | *serpyllum* | low | 17.02 | 1.2964 | 0.4 |
| Fabaceae | *Trifolium* | *badium* | high | 26.92 | 3.3166 | 0.9 |
|  |  | *pratense* | low | 24.32 | 2.4724 | 0.7 |


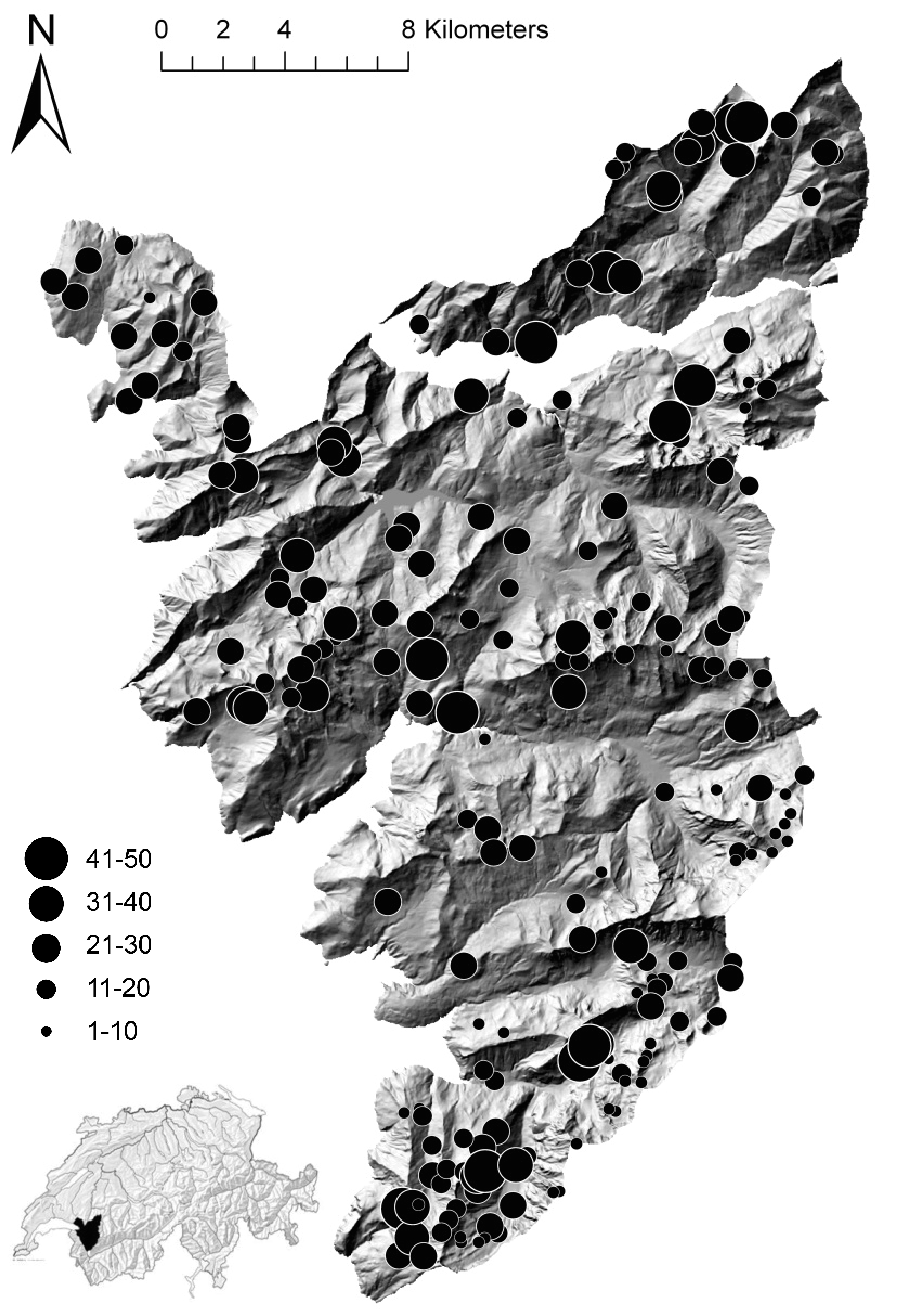


**Figure S1.** Study area in the Western Swiss Alps (indicated in black on the map of Switzerland). Black dots indicate sampled locations. Dot size is proportional to the number of butterfly species found at that location.


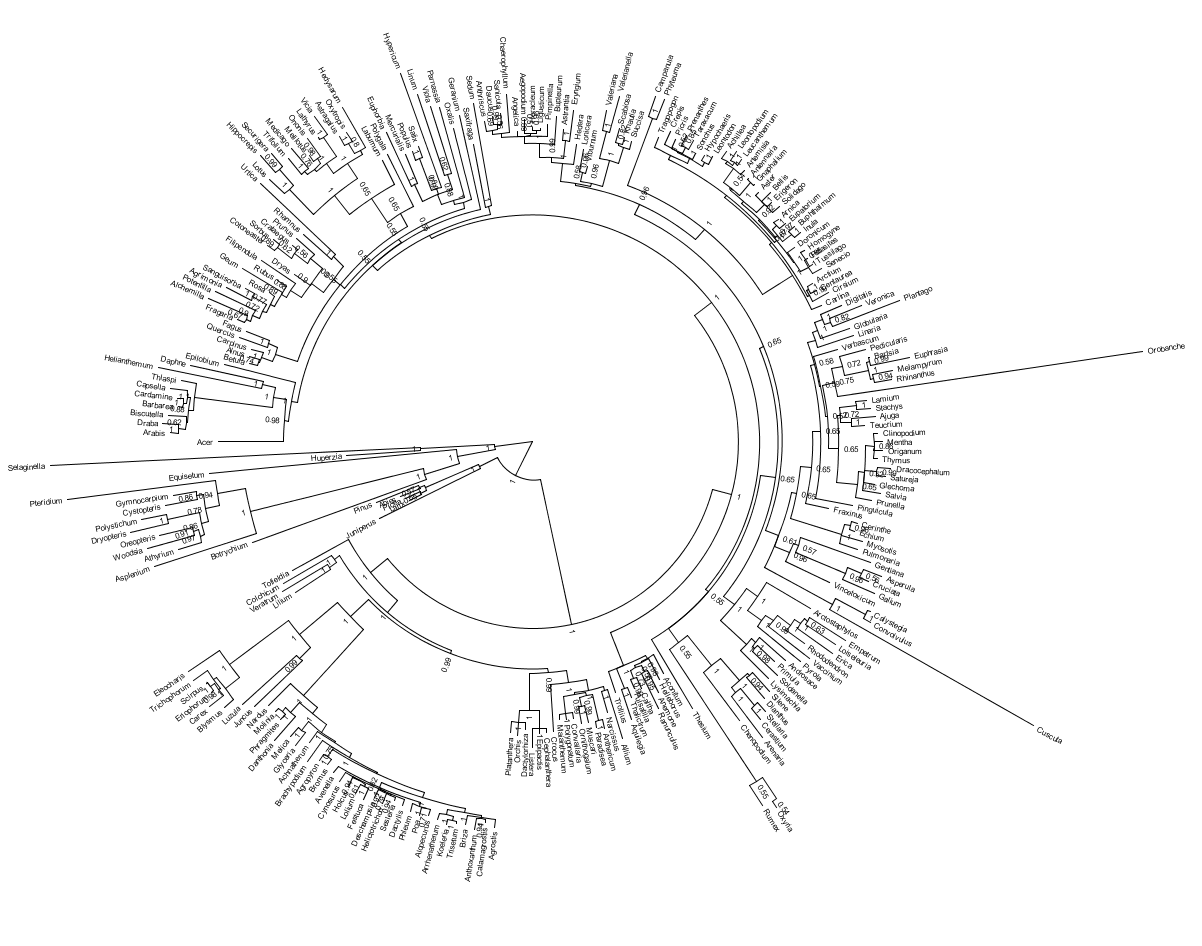


**Figure S2**. Angiosperm phylogeny. Shown is the consensus tree of the 256 plant genera found in the study area. The tree is rooted using genera from Pteridophyta and Gymnosperms occurring in the study area. The angiosperm genera span both monocotyledons and dicotyledons. Phylogenetic relationships were inferred using DNA sequences obtained from GenBank and including three chloroplast (ATP, RBCL, NDHF) loci. For both plant and butterflies’ groups (see Figure S2), sequences were aligned using MAFFT . MrBayes 3.1.2 was used to perform Bayesian analyses on the data. Models of sequence evolution for each region were calculated using MrModeltest 1.0 and were chosen based on the Akaike Information Criterion (AIC). A burn-in of 1,500 sampled generations was applied, and an all-compatible tree was reconstructed using the remaining 8,501 trees of each run (a total of 17,002 trees for the two runs), after which Bayesian posterior probabilities (BPP) were calculated.


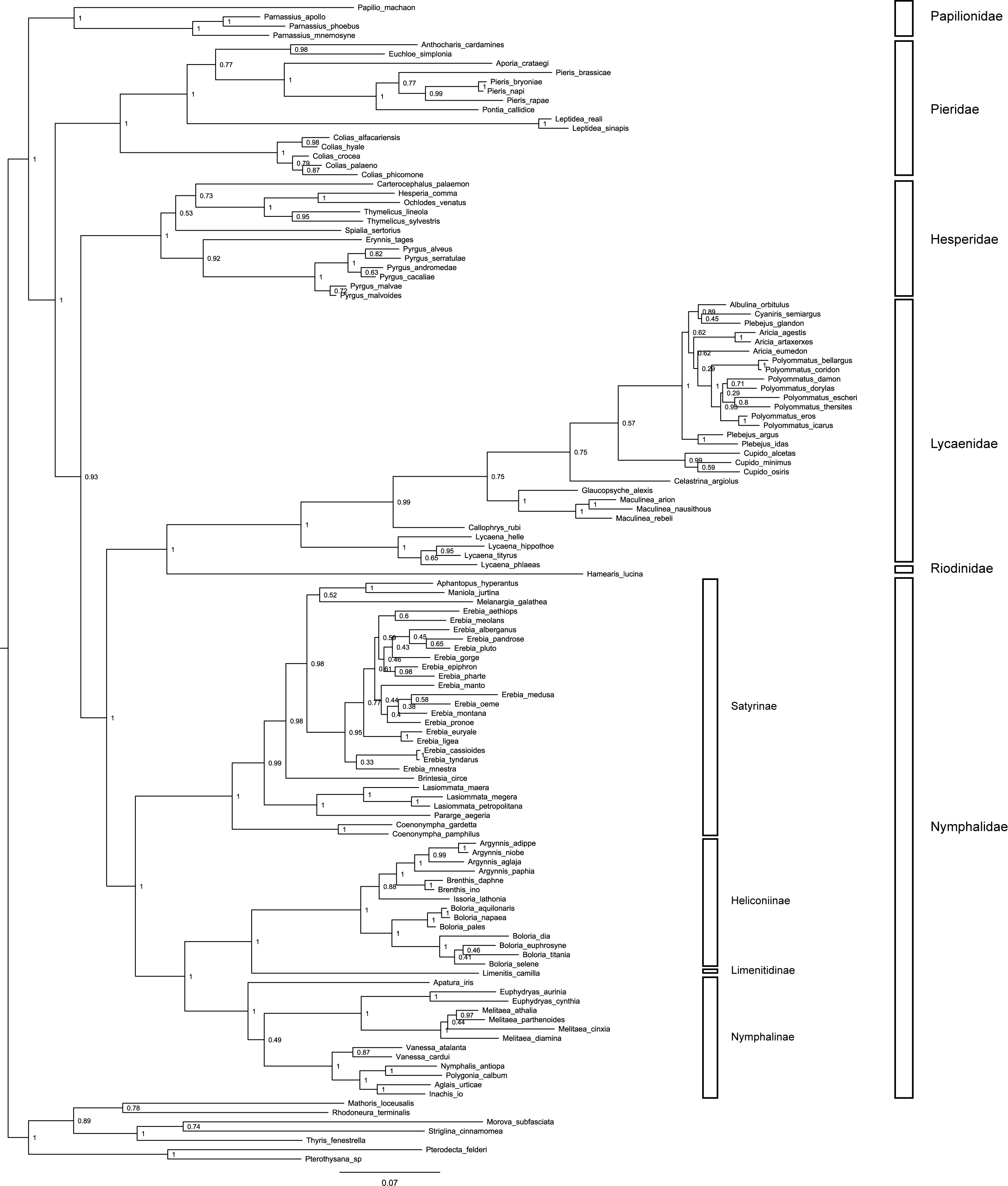


**Figure S3**. Butterfly phylogeny. Shown is the consensus tree of the 104 species of butterfly species found in the study area, belonging to the six families of butterflies, viz. Nymphalidae, Lycaenidae, Pieridae, Papilionidae, Riodinidae and Hesperiidae. Phylogenetic relationships were inferred using DNA sequences obtained from GenBank and including two nuclear markers (EF1-alpha, Wgl) and four mitochondrial markers (16s, COI, NDH1, NDH5). For sequence alignment and phylogenetic reconstruction see legend in Figure S1.

**
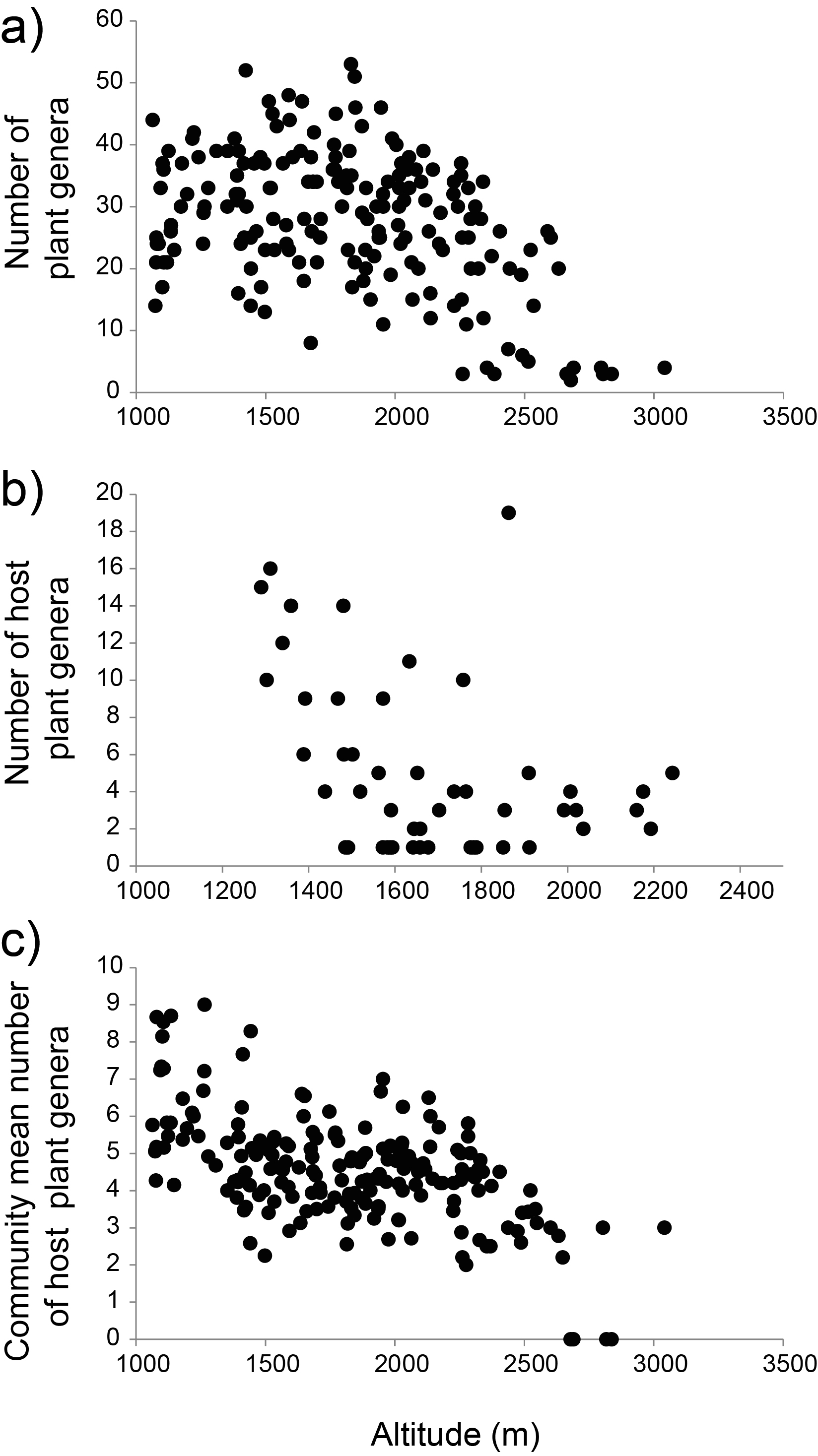
**

**Figure S4.** Relationship between the average elevation and a) the number of plant genera, b) butterfly’s diet breadth measured as total number of host plant genera consumed by each butterfly species, and c) the average number of genera consumed by species in the community. The number of plant genera decreases with altitude in non-linear fashion (linear, t = -7.6, p < 0.0001, and quadratic: t = -6.9, p < 0.0001). Plant genera used by butterfly species decreases with elevation, both for species (linear corrected for phylogenetic distance: df=47, F=4.5, p=0.039) and communities (linear: t =-7.93, p<0.0001, quadratic: t=1.5, p=0.35).


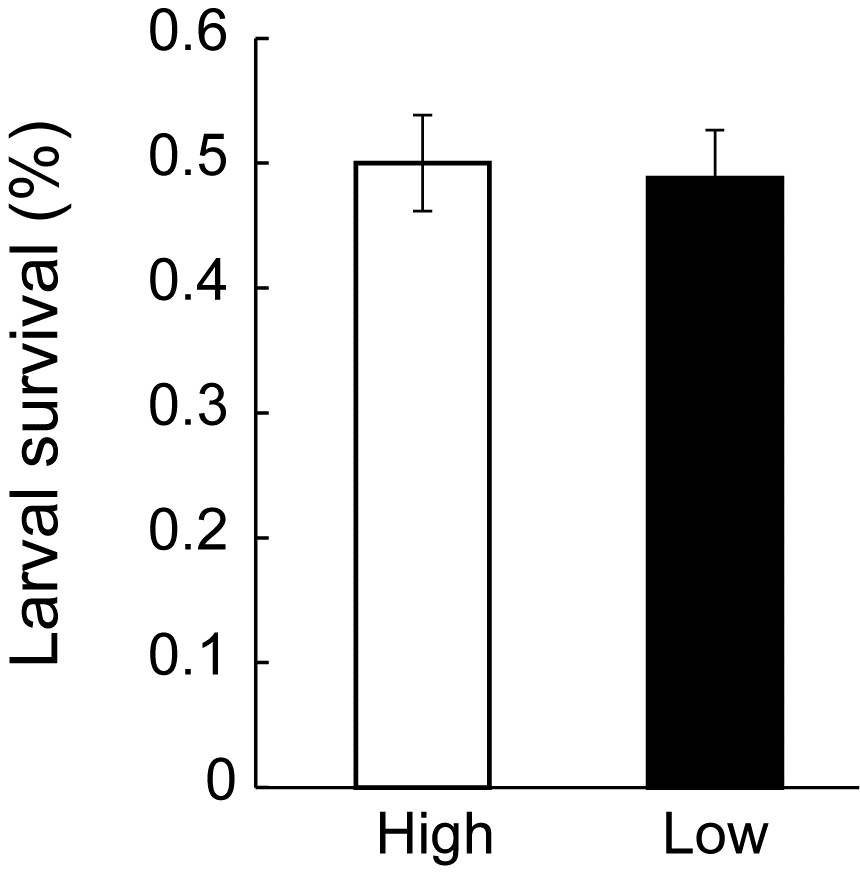


**Figure S5**. Larval survival on high and low elevation plants. Shown is the mean (± 1SE) of *Spodoptera littoralis* caterpillar survival on seventeen high elevation (open bars) plant species, and their congeneric low elevation (black bars) species. Plant species were randomly sampled along the phylogeny to include the most commonly found families (Table S1, Fig. 4).


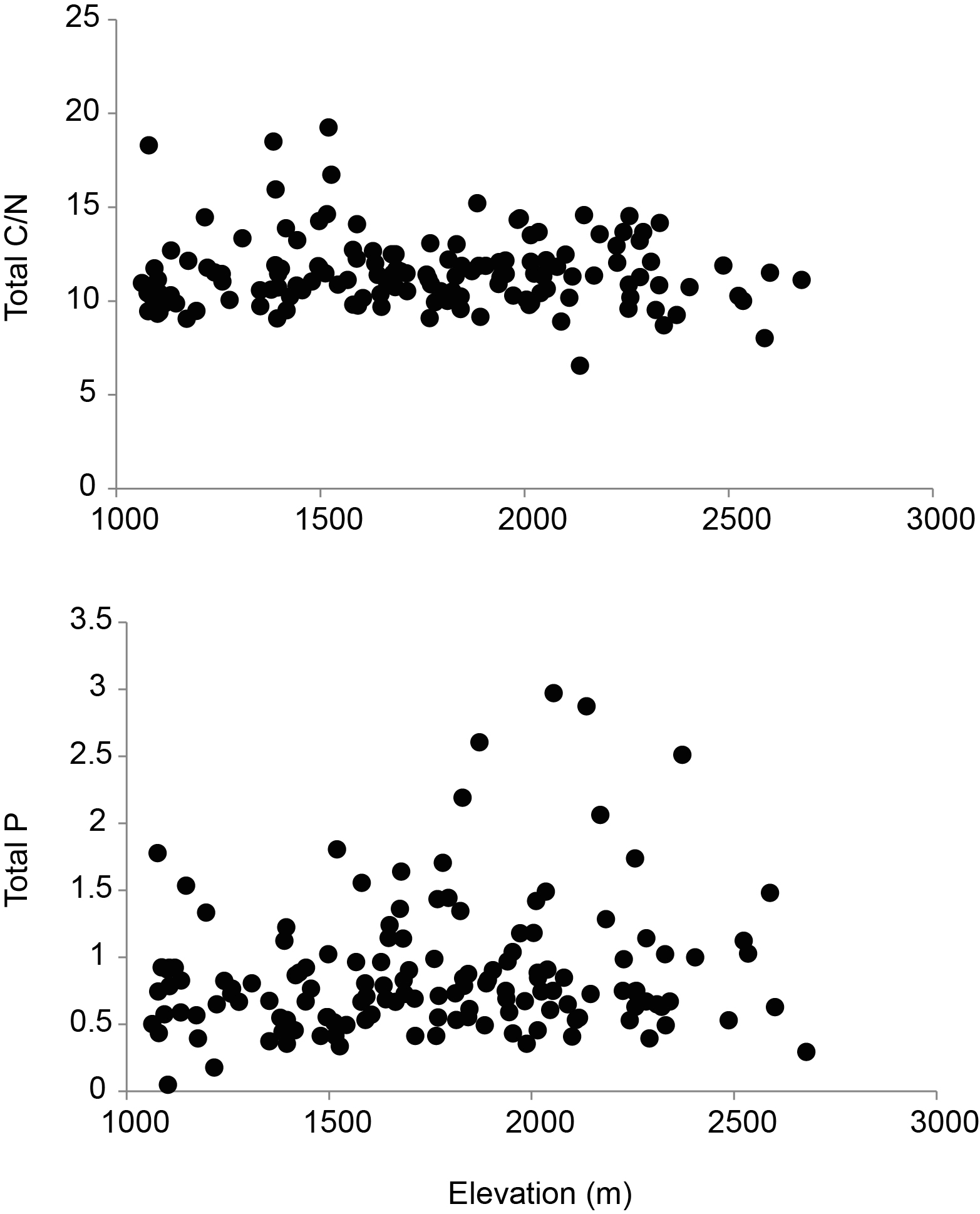


**Figure S6**. Soil nutrient composition was assessed by sampling the top 10 cm of the soil at each vegetation inventory. Soil samples were air-dried, sieved at 2 mm and grinded into powder. Nitrogen (N) and phosphorus (P) and organic carbon (C) content was measured with a CHN elementary analyzer. Soil C/N (df=141, F=0.39, p=0.53) and P (df=141, F=3.91, p=0.07) content did not change with elevation.


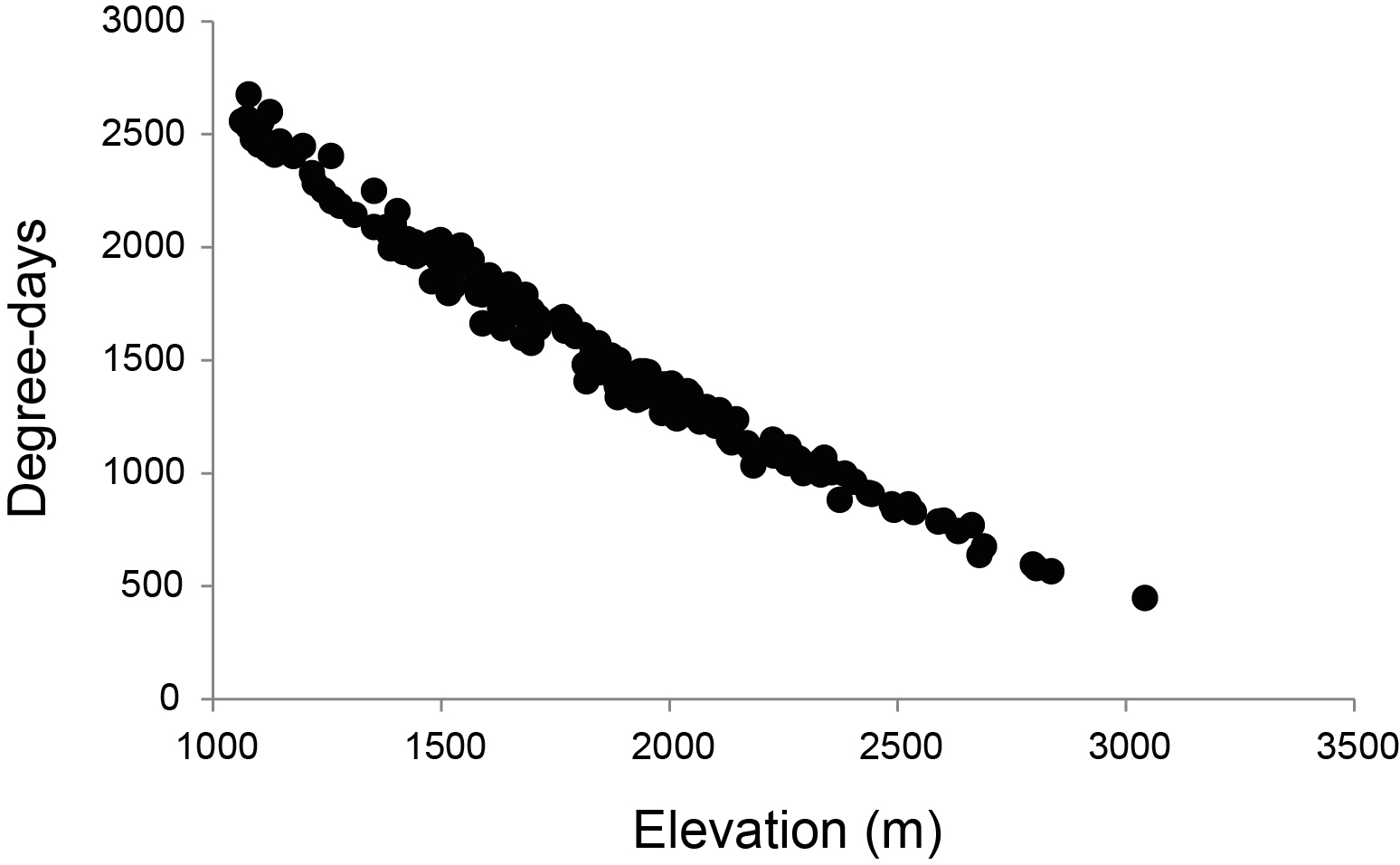


**Figure S7**. Number of degree-days along elevation gradients. Weather-related changes along elevation gradients were additionally assessed by relating degree-days above 0°C to elevation. The degree-day values were calculated from interpolated monthly average temperatures from a Swiss network of meteorological stations using the approach of Zimmermann and Kienast (1999). Correlation between degree-days and elevation was analyzed using LMs. Degree-days above 0°C decreased with elevation (df=141, F=6667, p<0.0001)

References

1. Katoh K, Misawa K, Kuma Ki, & Miyata T (2002) MAFFT: a novel method for rapid multiple sequence alignment based on fast Fourier transform. *Nucleic Acids Research* 30(14):3059-3066.

2. Ronquist F & Huelsenbeck JP (2003) MrBayes 3: Bayesian phylogenetic inference under mixed models. *Bioinformatics* 19(12):1572-1574.

3. Nylander JAA, Ronquist F, Huelsenbeck JP, & Nieves-Aldrey JL (2004) Bayesian phylogenetic analysis of combined data. *Systematic Biology* 53(1):47-67.
